# Activated Immune and Complement C3 Are Potential Contributors in MASH via Stimulating Neutrophil Extracellular Traps

**DOI:** 10.3390/cells14100740

**Published:** 2025-05-19

**Authors:** Ao Liu, Xiaoling Deng, Shuhui Hou, Yuwen Xi, Keshu Xu

**Affiliations:** Division of Gastroenterology, Union Hospital, Tongji Medical College, Huazhong University of Science and Technology, 1277 Jiefang Avenue, Wuhan 430022, China; xhdrla@hust.edu.cn (A.L.); dengxl2017@hust.edu.cn (X.D.); hsh030405@163.com (S.H.); xyw0563@hust.edu.cn (Y.X.)

**Keywords:** metabolic dysfunction-associated steatotic liver disease, complement C3, neutrophil extracellular trap, immunoglobulin, autoantibody

## Abstract

The number of metabolic dysfunction-associated steatotic liver disease (MASLD) patients is increasing rapidly. More attention has been paid to the relationship between immunity and MASLD. This study explored the roles of serum autoantibodies, immunoglobulins, and complements in MASLD. A total of 182 MASLD patients were investigated and grouped by autoantibody or NAS scores. Correlation between immunology and clinical features was assessed. In addition, metabolic dysfunction-associated steatohepatitis (MASH) models were constructed to verify the findings. Neutrophils were isolated from mice and treated with complement C3 to investigate the association between C3 and neutrophil extracellular traps (NETs). IgG, IgM, and NAS scores in the autoantibody positive group were significantly higher than those in the autoantibody negative group. Antinuclear antibodies (ANA), IgA, IgE, IgG, C3, C4, ALT, and AST were related to MASH. Meanwhile, IgA and C3 correlated with the severity of MASLD. The ROC curve showed that IgA > 2.990 g/L or C3 > 1.115 g/L predicted the presence of MASH. More importantly, IgG, activated C3, and NETs were increased in MASH. C3 stimulation directly induced NET formation in the neutrophils. Immunity systems were activated in MASH and elevated IgG activated C3 to stimulate the production of NETs, thus exacerbating MASH.

## 1. Introduction

Metabolic dysfunction-associated steatotic liver disease (MASLD) is a group of diseases characterized by abnormal liver metabolism [1,2]. It can be histologically divided into simple fatty liver, metabolic dysfunction-associated steatohepatitis with or without fibrosis (MASH), and metabolic dysfunction-associated cirrhosis. A recent meta-analysis of MASLD showed that the global prevalence of MASLD is 39% [3]. Moreover, it is expected to affect 314.58 million people by 2030 [4]. These alarming statistics underscore the urgency of understanding MASLD pathogenesis to reduce its societal burden. Early recognition and mechanistic insights into disease progression are particularly critical to develop targeted interventions.

Beyond metabolic dysfunction, immune dysregulation is increasingly recognized as a contributor to MASLD pathogenesis. The most widely applied serum autoantibody, antinuclear antibody (ANA), has been observed in serum of 16.9–43% MASLD patients after excluding autoimmune liver disease [5,6] and it is independently associated with stage F3-4 liver fibrosis [7]. Although the correlation between autoantibodies and MASLD remains unclear, adaptive immune responses have been found to promote the progression of MASLD [8].

In addition to autoantibodies, hepatic immune activation may further modulate disease progression through immunoglobulin overproduction. For instance, a prospective study showed that elevated immunoglobulin G (IgG) was a significant predictor of hepatic decompensation in MASH patients [9], while immunoglobulin M (IgM) appeared to ameliorate chronic inflammation of the liver and insulin resistance [10,11]. These opposing roles of immunoglobulins highlight the complexity of immune-mediated mechanisms in MASLD, yet studies correlating autoantibodies and immunoglobulins with the progression of MASLD are still insufficient.

Alongside adaptive immunity, emerging evidence suggests that the complement system is critically involved in MASLD [12]. MASLD patients exhibit high levels of complement C3, which is positively correlated with the severity of fatty liver [13]. A cross-sectional study of 4729 participants concluded that C3 is a risk factor for MASLD progression [14]. This complement-driven inflammatory cascade may intersect with other immune pathways to exacerbate liver injury.

Recent studies propose that neutrophil extracellular traps (NETs), fibrous networks extruded by activated neutrophils, could serve as a mechanistic bridge between complement activation and tissue damage [15]. NETs, composed of histones and depolymerized chromatin fibers, are implicated in aberrant immune responses when overproduced or inadequately cleared [16]. Notably, prior work has linked NETs to complement system activation [17], but their role in MASLD has not been thoroughly studied.

To address these knowledge gaps, this study systematically evaluated alterations in serum autoantibodies, immunoglobulins, and complement components in MASLD patients, correlating these immune biomarkers with clinical progression or histological severity. Using a murine MASLD model, we further demonstrated that IgG-driven C3 activation triggers NET formation to exacerbate MASH. Our findings not only identify potential biomarkers for risk stratification but also highlight therapeutic opportunities targeting complement signaling or NETs pathways to mitigate MASLD progression.

## 2. Materials and Methods

### 2.1. Participants

A total of 182 MASLD patients who were hospitalized for abnormal liver function between January 2019 and July 2024 at a tertiary hospital in Wuhan were surveyed in this retrospective cross-sectional study. The inclusion criteria were as follows: (1) b-ultrasonography, CT, MRI or liver biopsy showed fatty liver; (2) the results of serum autoantibodies, immunoglobulins, and complements were preserved completely. The exclusion criteria included: (1) alcohol consumption of >30 g/day in men and >20 g/day in women; (2) autoimmune hepatitis patients and other patients with a simplified diagnostic criteria score ≥ 6 for autoimmune hepatitis [18]. Notably, in clinical practice, liver biopsies were performed to confirm the presence of autoimmune hepatitis (AIH) in patients with suspected AIH or a simplified score of 6. If there was only mild elevation of antibodies such as ANA and no elevation of IgG, the patient was not considered to have AIH; (3) patients with other autoimmune diseases (like Hashimoto); (4) patients with other diseases causing hepatic steatosis (viral hepatitis, autoimmune liver disease, drug-induced liver disease, hereditary liver disease, etc.); (5) patients with malignant comorbidities.

As soon as patients were admitted to the hospital, they underwent an initial examination, which included the collection of a blood sample and other matters. The results of this pre-treatment examination were collected. Specifically, age, sex, body mass index (BMI), comorbidity (hypertension, diabetes), biochemical markers (ALT, AST, LDL, HDL, serum calcium, uric acid, creatinine, urea nitrogen), autoimmune antibodies (ANA, smooth muscle antibody (SMA), anti-mitochondrial antibody (AMA), liver–kidney microsome antibody (LKM) and others), immunoglobulins (IgA, IgG, IgE, IgM), complements (C3, C4), liver b-ultrasound, CT, MRI, FibroTouch [19], and liver biopsy pathology were collected.

In this study, ANA was detected via indirect immunofluorescence. A dilution titer of 1:100 and above was considered ANA positive, based on its widespread adoption in Chinese diagnostic guidelines [20,21,22]. Autoantibody positivity was defined as being positive for any of the following: ANA, SMA, AMA, anti-Smith antibody, anti-Scl-70 antibody, anti-SS(A) antibody, anti-SS(B) antibody, anti-Jo-1 antibody, anti-dsDNA antibody, anti-ribosomal P-protein antibody, anti-centromere protein B antibody, anti-ribonucleoprotein A antibody, anti-ribonucleoprotein 68 antibody, anti-chromatin antibody, anti-Ro 52 antibody, anti-M2-3E antibody, anti-sp100 antibody, anti-gp210 antibody, anti-liver cytosolic antigen type 1 antibody, and anti-soluble liver antigen antibody. Autoantibody negativity was defined as being negative for all of the autoantibodies tested above. This study was approved by the local Ethics Committee of the Union Hospital, Tongji Medical College, Huazhong University of Science and Technology (Approval Number: UHCT230148) and performed in accordance with the ethical standards laid down in the 1964 Declaration of Helsinki and its later amendments. Informed consent of patients was waived by the local Ethics Committee in view of the retrospective nature of the study and details that might disclose the identity of the subjects under study were omitted.

### 2.2. Animals

Six-week-old male C57BL/6J mice were obtained from the Shulaibao Animal Center (Wuhan, China). All animals were raised in the experimental animal center of the Tongji Medical College, Huazhong University of Science and Technology. The protocol and procedures employed were ethically reviewed and approved by the local Experimental Animal Ethics Committee of Huazhong University of Science and Technology (IACUC number: 4399). All animals were housed in a controlled environment (25 °C, 12 h light/dark cycle), and had ad libitum access to tap water and food. The mice were fed a Gubra amylin NASH diet (GAN diet; 40 kcal% fat, 22% fructose, 10% sucrose, 2% cholesterol; D09100310, Research Diets, New Brunswick, NJ, USA) for 24 weeks, resulting in MASH, while the control mice were fed a normal standard diet. The body weight was recorded twice a week. The blood was collected from the inferior vena after execution.

### 2.3. Cell Culture and Treatment

Mouse liver cells from line AML-12 (CL-0602; Pricella, Wuhan, China) were grown in DMEM/F12 supplemented with 10% FBS, 1% ITS (I3146; Sigma, St. Louis, MO, USA), 100 U/mL of penicillin, and 100 µg/mL of streptomycin at 37 °C in 5% CO_2_. STR identification was correct to ensure the authenticity of the cells. Free fatty acid (FFA) mixtures with low proportions of palmitic acid (oleate/palmitate, 2:1) showed mild toxicity and apoptotic effects [23]. To mimic benign chronic steatosis, 400 μM oleic acid (OA) and 200 μM palmitic acid (PA) were added to treat the cells for 48 h [24]. Then, the cells were harvested for further analysis.

### 2.4. Neutrophils Isolation and Intervention

Neutrophils were isolated from murine bone marrow via immunomagnetic negative selection following kit protocols (#19762; Stemcell, Vancouver, BC, Canada). Briefly, mice were euthanized via cervical dislocation, and bilateral tibiofemoral bones were immediately dissected. Bone marrow cells were flushed from the medullary cavity after transecting both bone ends. Non-target cells were labeled with biotinylated antibodies and magnetic beads, followed by magnetic separation to enrich neutrophils. A subset of isolated neutrophils underwent Wright–Giemsa staining to validate purity (>90%), while the remaining cells were immediately utilized for downstream assays.

Isolated neutrophils were treated with complement C3 (200 nM; HY-P7863; MCE, Monmouth Junction, NJ, USA) or vehicle control (equivalent volume of culture medium) for 24 h. For immunofluorescence studies, neutrophils were seeded onto chamber slides, pre-coated with poly-L-lysine (0.5 mg/mL), 30 min prior to C3 treatment to ensure optimal cellular adhesion.

### 2.5. Histological Analysis

Liver specimens were fixed in 4% paraformaldehyde for 48 h, embedded in paraffin, and cut into sections subsequent to hematoxylin-eosin (HE) staining and immunohistochemistry assay according to standard protocols. Morphological changes were then digitalized using an Olympus light microscope by another experimenter. The histological changes of liver were assessed by the NAS score based on the MASH CRN scoring system [25]. Antibodies used in immunohistochemistry were anti-c3 (sc-28294; Santa Cruz, Dallas, TX, USA), and anti-MPO (myeloperoxidase, GB15224; Servicebio, Wuhan, China).

### 2.6. PCR

Total RNA was extracted from tissue by TRIZOL according to the manufacturer’s protocol, and cDNA was synthesized by SuperMix (#R323, Vazyme, Nanjing, China). Then, real-time fluorescence quantitative PCR was operated with SYBR Green Master Mix (#Q111, Vazyme, China) in the Roche Lightcycle R480. The primer sequences are listed in Supplementary Table S1. The expression of target genes was analyzed by 2^−ΔΔCt^ method.

### 2.7. Western Blot

Total proteins were extracted from tissue and then quantified by the BCA Kit (Beyotime, Shanghai, China). Different proteins were separated on SDS-PAGE gel and transferred to a PVDF membrane. Fractionated proteins were probed overnight with primary antibody at 4 °C, then incubated with the secondary antibody for 1 h at room temperature. The immunoblots were visualized by a ECL chemiluminescence detection kit (Vazyme, Nanjing, China). Antibodies: anti-c3 (sc-28294; Santa Cruz), anti-citrullinated histone H3 (citH3) antibody (ab5103 and ab281584; Abcam, Cambridge, UK), anti-GAPDH antibody (ANT325; Antgene, Wuhan, China), goat anti-mouse IgG (ANT019; Antgene), and goat anti-rabbit IgG (ANT020; Antgene).

### 2.8. Immunofluorescent Staining

The tissues were embedded in paraffin and sliced, then the slides were lysed, blocked, and incubated with the primary antibody. After that, slices were incubated with secondary antibodies. We measured NETs specifically identified by positive staining of MPO and citH3 [26,27]. Antibodies: anti-c3 (sc-28294; Santa Cruz), anti-citH3 antibody (NB100-57135; Novus and ab5103; abcam), anti-MPO antibody (66177-1-Ig; Proteintech Wuhan, China and GB15224; Servicebio) and DAPI (G1407; Servicebio). Secondary antibodies: Cy3-conjugated anti-rabbit IgG (1:200; Servicebio) and Alexa Fluor 488-conjugated anti-mouse IgG (1:200; Servicebio).

### 2.9. ELISA

The serum was obtained from the mice blood samples by centrifugation at 3500 g for 15 min. Then, protein levels of C3, C3a, and IgG were measured by ELISA (E-EL-M0330 and E-EL-M0337, Elabscience, Wuhan, China; EMC116, Neobioscience, Shenzhen, China) according to the manufacturer’s instructions.

### 2.10. Statistical Analyses

The selected cases were divided into two groups according to autoimmune antibodies and NAS score. Then, the differences of demographic characteristics, laboratory examination, and pathological examination were compared. Using the presence of MASH as outcome variables, logistic regression was used for univariate and multivariate analysis. Receiver operating characteristic (ROC) curves were drawn with significant variables.

Categorical variables are shown as percentages, and comparisons between two groups were analyzed by chi-square test. Continued variables are shown as mean ± standard error of mean (SEM) and were analyzed by t-test or one-way ANOVA. A two-way ANOVA with Tukey’s multiple comparisons test was performed for multivariate analysis. Data were analyzed by SPSS 25.0 and GraphPad Prism 8. The schematic model was created in Adobe Illustrator 2023. A *p* value < 0.05 was considered statistically significant.

## 3. Results

### 3.1. Demographic and Clinical Characteristics

A total of 182 MASLD patients were included in this study. Demographic and clinical characteristics such as comorbidity, autoantibodies, immunoglobulin and complement levels were recorded (Supplementary Table S2). The average age of the population was 42.4 years, and 57.1% of the patients were male. The median BMI of the population was 25.8, indicating patients were overweight in general. Notably, 36.8% MASLD patients had at least one positive autoantibody test, and ANA was the most common positive autoantibody, accounting for 31.3% of the entire study cohort.

The mean or median values of IgA, IgG, IgM, IgE, complement C3, and C4 in all MASLD populations were within the normal range. The same was true for biochemical tests, blood lipids, PT, APTT, red blood cell count (RBC) and hemoglobin. Nevertheless, the median of controlled attenuation parameter (CAP) and liver stiffness measurements (LSM) exceeded normal values, indicating the prevalence of fatty liver and mild liver fibrosis in these patients.

Pathologic data were available for 86 of the 182 MASLD patients. The results of hepatic steatosis, hepatocyte ballooning, and intralobular inflammation are shown (Supplementary Table S3). The NAS score of liver was 3.2 ± 1.8, and 24.4% (21/86) patients were diagnosed with MASH. Furthermore, the percentages of patients with hepatic fibrosis scores F0, F1, F2, F3, and F4 were 44.2%, 32.6%, 12.8%, 3.5%, and 7.0%, respectively.

### 3.2. Comparison of Characteristics Between MASH Group and Non-MASH Group

Next, the study population was divided into MASH group and non-MASH groups according to whether the NAS was ≥5. The ratio of ANA positivity was higher in the MASH group than that in the non-MASH group (66.7% vs. 41.5%; *p* = 0.045; Table 1). IgA, IgE, and IgG values in the MASH group were higher than those in the non-MASH group (*p* = 0.023, 0.069, and 0.353).

Importantly, C3 (*p* = 0.007) and C4 (*p* = 0.039) were also higher in the MASH group than in the non-MASH Group. In addition, the levels of ALT and AST in the MASH group were significantly higher than those in the non-MASH group (*p* < 0.05). It is suggested that ANA, IgA, IgE, IgG, complement C3, C4, ALT, and AST may be related to the higher-grade histological manifestations.

### 3.3. Comparison of Features Between Autoantibody Negative Group and Autoantibody Positive Group

Patients were divided into two groups according to whether their autoantibody tests were positive or not. The positive group had more female patients than the negative group (*p* < 0.05, Supplementary Table S4). The levels of IgG and IgM in the positive group were significantly higher than those in the negative group (*p* < 0.05).

The pathological results of liver biopsy were also compared between the two groups. The NAS score in the positive group was significantly higher than that in the negative group (*p* = 0.031, Table 2). Autoantibody-positive patients exhibited a trend towards a higher proportion of MASH diagnosis (31.3% vs. 15.8%, *p* = 0.097), but this did not reach statistical significance, likely due to limited subgroup sizes.

### 3.4. Correlation Analysis Between Immunological Indicators and Liver Histology

Univariate and multivariate analysis were performed by logistic regression, using histologic characteristics as outcome variables. The results of univariate analysis showed IgA, C3, and ALT might be the risk factors for steatosis (>33%, Supplementary Table S5), ANA and C3 might be the risk factors for ballooning, and ANA, IgA, and C3 may be the risk factors for NAS (≥5, Supplementary Table S6). However, no variables were associated with lobular inflammation. Multivariate analysis showed that IgA and C3 were independent risk factors for NAS (≥5; Table 3).

### 3.5. The Predictive Value of Immunological Indicators for MASH

Based on the results of multivariate analysis, ROC curves were plotted and their predictive value was compared (Supplementary Figure S1). The area under curve (AUC) of C3 for predicting NAS (≥5) was 0.752 (95% CI: 0.593–0.911, *p* = 0.002). The optimal cutoff value was 0.968 g/L. The sensitivity and specificity were 76.5% and 75.0%, respectively. For IgA, the AUC for predicting NAS (≥5) was 0.657 (95% CI: 0.500–0.815, *p* = 0.055), and the cutoff value was 2.990 g/L. The sensitivity and specificity were 47.1% and 85.4%. Additionally, the AUC for the combined prediction of NAS (≥5) by IgA and C3 was 0.783 (95% CI: 0.641–0.925, *p* = 0.001). C3 had a greater predictive value than IgA. The AUC combining C3 with IgA only rose from 0.752 to 0.783. Thus, further research focused on the role of C3 in MASH.

### 3.6. Elevated IgG and Complement C3 in MASH

**Figure 1:**
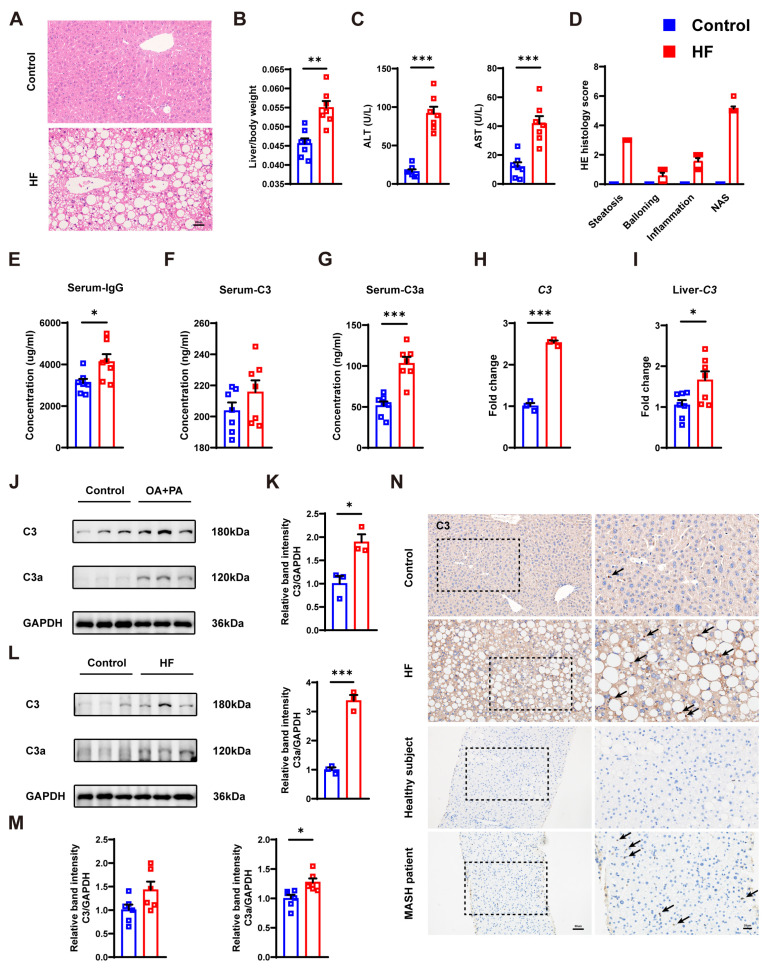
HF diet-fed mouse model showed increased levels of IgG, C3, and C3a. (**A**) Representative HE images of mouse liver, bar: 50 μm. (**B**) Liver/body weight of control and HF diet-fed group. (**C**) ALT and AST of two groups. (**D**) HE histology scores of two groups. (**E**–**G**) Serum lgG, C3, and C3a of two groups. (**H**,**I**) mRNA levels of C3 in the cell and mouse model. (**J**–**M**) Western blots and the relative band intensity of C3 and C3a in two models. (**N**) Representative immunohistochemistry images of C3 in the liver of mouse, healthy subjects, and MASH patients. Arrows exhibit positive staining. Bar left and right: 50 μm and 20 μm. * *p* < 0.05, ** *p* < 0.01, *** *p* < 0.001.

**Figure 2:**
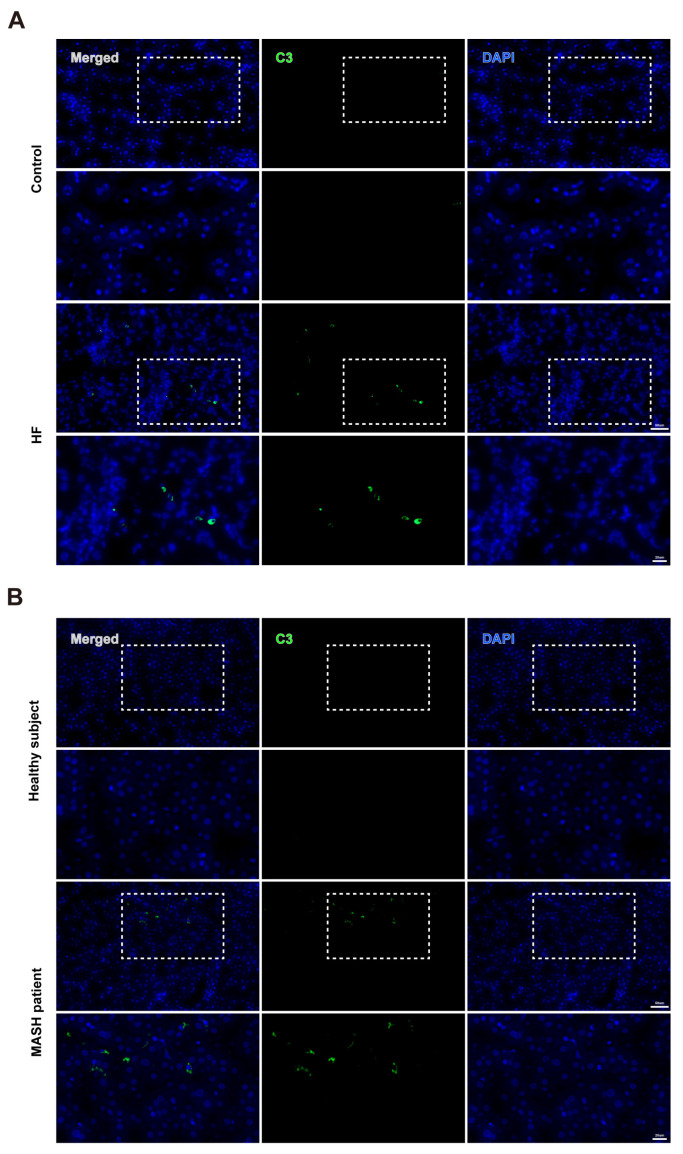
Immunofluorescence images of C3 in the liver of mice and patients. (**A**) Representative immunofluorescence images of C3 in the control and HF diet-fed mice. (**B**) Representative immunofluorescence images of C3 in healthy subjects and MASH patients. Bar above and below: 50 μm and 20 μm.

We further explored the relationship between C3 and MASH in the MASH animal model (Figure 1A–D). Serum IgG and C3 were increased in the high-fat (HF) diet-fed group detected by ELISA (Figure 1E,F). Meanwhile, C3a, an active fragment of C3 that can elicit acute inflammatory reactions, was also elevated in the HF group (Figure 1G). PCR showed the level of C3 was increased in the OA+PA-treated cell model and HF diet-fed mouse (Figure 1H,I). Furthermore, similar results were observed for protein expression levels of C3 and C3a (Figure 1J–M). The results of immunohistochemistry and immunofluorescence also showed increased C3 in the HF diet-fed group and the MASH patients (Figure 1N and Figure 2).

### 3.7. Production of NETs Increased in MASH

**Figure 3:**
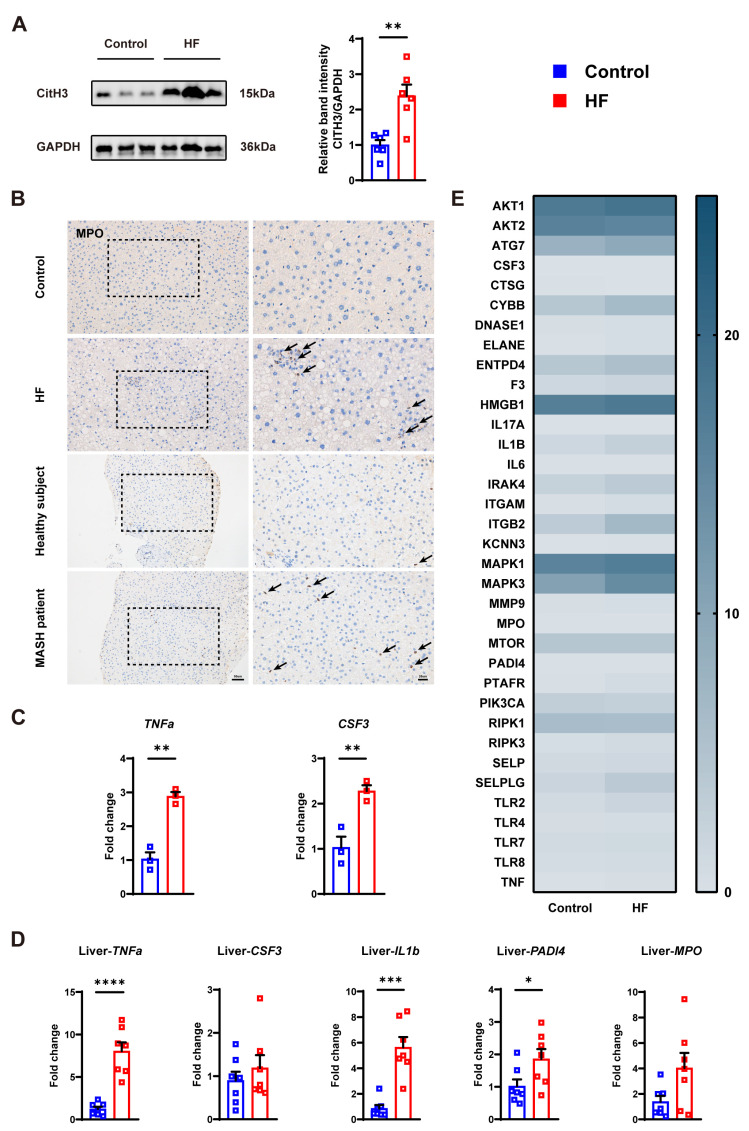
Detection of NETs in MASH. (**A**) Western blot of citH3 in the mouse model. (**B**) Representative immunohistochemistry images of MPO in the liver of mouse, healthy subjects, and MASH patients. Arrows exhibit positive staining. Bar left and right: 50 μm and 20 μm. (**C**) mRNA levels of TNFa and CSF3 in the cell model. (**D**) mRNA levels of TNFa, CSF3, IL1b, PADI4, and MPO in the mouse liver. (**E**) A series of NET-related genes in the RNA sequencing of mouse liver. * *p* < 0.05, ** *p* < 0.01, *** *p* < 0.001, **** *p* < 0.0001.

Previous studies had shown that the complement C3 may promote NET activation [17,28]. Therefore, we further detected the production of NETs. MPO is found primarily in the aspergillus granules of neutrophils and is released extracellularly by degranulation when neutrophils are stimulated. During the formation of NETs, histone H3 is citrullinated to citH3 by peptidyl arginine deiminase 4 (PADI4) at residues R2, R8, and R17. Subsequently, citH3, together with MPO, is embedded in the DNA backbone, forming specialized extracellular network structures, NETs. Thus, Western blotting of citH3 was performed and we found increased expression of citH3 in the HF diet-fed group (Figure 3A). Immunohistochemistry also showed more MPO in the HF group and MASH patients (Figure 3B). In addition, an inflammatory factor, tumor necrosis factor a (TNFa), was increased in the OA+PA treated group. TNFa and interleukin-1b (IL1b) were elevated in the HF diet-fed group (Figure 3C,D). As expected, NET-related gene colony-stimulating factor 3 (CSF3) was increased in the OA+PA treated group. CSF3, PADI4, and MPO were increased in the HF group. Moreover, RNA sequencing of mouse liver revealed elevated expression of most NET-related genes in the HF group relative to controls (Figure 3E, Supplementary Table S7). Notably, the HF group displayed significant upregulation of ELANE, IL6, IRAK4, PTAFR, RIPK3, SELP, and TNF (*p* < 0.05).

**Figure 4:**
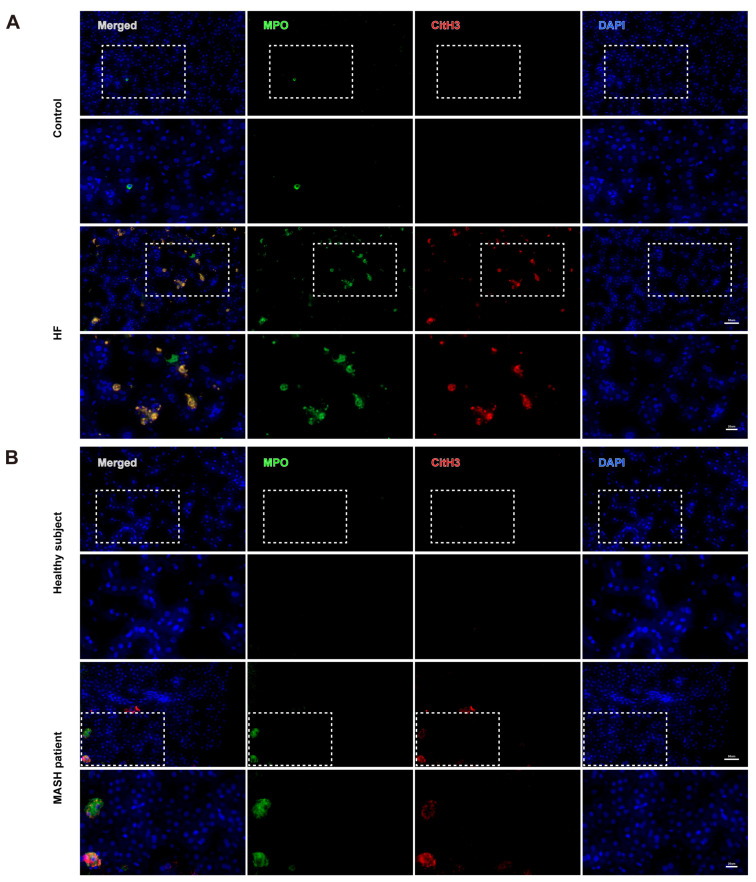
Immunofluorescence images of NETs in the liver by positive co-staining of MPO and citH3. (**A**) Representative immunofluorescence images of NETs in the control and HF group. (**B**) Representative immunofluorescence images of NETs in healthy subjects and MASH patients. Bar above and below: 50 μm and 20 μm.

Subsequently, NETs were specifically identified in immunofluorescence experiments by positive co-staining of MPO and citH3 (Figure 4A,B). The HF diet-fed group and MASH patients also had more positive co-staining rates than the control group and healthy subjects.

### 3.8. C3 Stimulation Induces NET Formation in the Neutrophils

**Figure 5:**
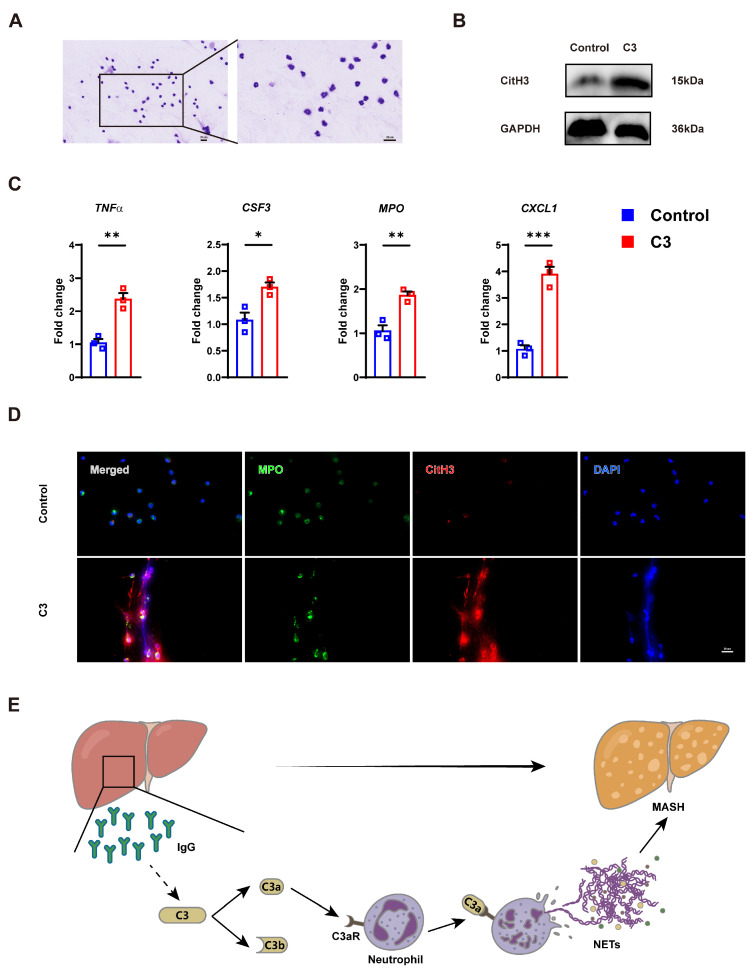
C3 induces the production of NETs in neutrophils, and the schematic model. (**A**) Representative images of Wright–Giemsa staining of neutrophils. (**B**) Western blot of citH3 in neutrophils following C3 intervention. (**C**) mRNA levels of TNFa, CSF3, MPO, and CXCL1 in neutrophils following C3 intervention. (**D**) Representative immunofluorescence images of NETs in neutrophils following C3 intervention. (**E**) The schematic model: elevated IgG activates C3 to stimulate the production of NETs, thus exacerbating MASH. * *p* < 0.05, ** *p* < 0.01, *** *p* < 0.001.

Neutrophils were isolated from murine bone marrow via immunomagnetic negative selection and Wright–Giemsa staining was used to validate purity (>90%) of isolated neutrophils (Figure 5A). After treatment with complement C3 or vehicle control, increased expression of citH3 was found in the C3-treated group (Figure 5B). Inflammatory factor TNFa and NET-related genes CSF3, MPO and C-X-C motif chemokine ligand 1 (CXCL1) were also elevated (Figure 5C). Notably, complement C3 stimulation significantly enhanced NET formation, as evidenced by increased extracellular DNA-MPO-CitH3 colocalization (Figure 5D). Collectively, these findings demonstrate that C3 acts as an inducer of NETs in neutrophils, providing mechanistic evidence for its role in amplifying inflammatory cascades.

## 4. Discussion

The prevalence of MASLD has increased rapidly in recent years. More importantly, the number of hospitalizations caused by MASLD-related end-stage liver disease is also significantly increased [29]. To stop this trend, the present study investigated the association of serum autoantibodies, immunoglobulins, and complements with the histologic features of MASLD, which may provide new ideas for early intervention in MASLD, decreasing the incidence of end-stage liver disease and reducing the disease burden.

Among 182 non-AIH patients with MASLD, 67 (36.8%) patients were positive for at least one autoimmune antibody, and 57 (31.3%) patients showed ANA positivity, which is similar to previous reports [30,31]. Then, patients were divided into two groups according to whether they were positive for autoantibodies. The positive group had more female patients than the negative group (65.7% vs. 29.6%, *p* < 0.001), which was consistent with the prevalence of autoimmune hepatitis in women [32]. This was possibly related to sex hormones, which could promote expanded T/B cells overactivation and autoantibody production, but it may also be associated with lifestyle and environmental differences brought about by gender differences [33].

Additionally, in agreement with other studies [7,34], IgG, IgM, and NAS score were statistically different between the two groups, indicating that immune activation is involved in the pathogenesis of MASH. A study analyzing 388 MASLD patients found that ANA positivity was independently associated with stage F3-4 liver fibrosis [7]. In contrast, other studies showed no difference between the two groups in the presence of cirrhosis and MASH after grouping by ANA positivity [6,35]. A possible explanation for this might be that the dilution titers that defined ANA positivity were different.

To further clarify the relationship between immune activation and liver histology, the study population was divided into MASH and non-MASH groups. The results showed that the MASH group presented a high rate of ANA positivity compared with the non-MASH group, but no difference in the titer of ANA was presented between the two groups. More studies on ANA with different titers and MASLD are suggested.

In addition, in line with other study [36], the comparison of immunoglobulin and complement in MASH and non-MASH groups revealed that IgA, C3, and C4 were associated with MASH. IgE and IgG values in the MASH group were higher than those in the non-MASH group but had no significant difference (*p* = 0.069, and 0.353). This may be due to an insufficient sample size. Furthermore, IgG1 and IgG3 strongly activate the classical complement pathway due to their flexible hinge regions and high C1q affinity [37]. Subclass-specific changes (e.g., IgG3 dominance) may drive C3 activation even if total IgG remains unchanged [38,39]. Immunoglobulin subtypes, especially IgG3, are a critical focus for future studies. Thus, the results suggested that ANA, immunoglobulin, C3, and C4 played an important role in facilitating the progression of MASLD to MASH.

Further, univariate analysis showed that IgA, ANA, and C3 were significant variables for the MASH histological characteristics. Nevertheless, only IgA and C3 were independent risk factors for NAS (≥5) in the multivariate analysis. IgA is a major part of the mucosal defense system, and intestinal dysbacteriosis plays an important role in the progression of MASLD. By comparing IgA in patients with early and advanced MASLD, studies have shown that increased IgA is an independent risk factor for liver cirrhosis [40]. By contrast, another cross-sectional study found that IgA did not differ between the MASH and non-MASH groups (3.5 ± 1.2 vs. 2.9 ± 0.8, *p* = 0.052), but it was also positively associated with liver fibrosis [41]. B cell activation in the systemic or gastrointestinal tract promoted IgA production, which may activate hepatic myeloid cells through an IgA-FcR signaling axis to induce hepatic fibrosis [42].

Numerous components of the complement system, including C3, are primarily produced by the liver. The complement system is a key part of liver homeostasis, and C3 has been found to promote hepatic steatosis and obesity-related inflammation [43]. Consistent with previous findings [14,44,45], serum C3 was higher in MASLD patients than in healthy subjects in this study. The presence and severity of MASLD were linked with the serum C3 level, which was an independent risk factor for the diagnosis of MASLD [46]. Moreover, ROC curves showed C3 had better predictive value of NAS (≥5) than IgA (0.752 vs. 0.657). Despite combining C3 with IgA, the AUC only rose from 0.752 to 0.783. Thus, the next step focused on investigating the role of C3 (rather than IgA) in MASH.

We first noted that the complement system was physiologically functional only when it was activated. There are three pathways to activate it [47]: the lectin pathway plays a role in the early anti-infection process and in addition requires pathogens as activators. Alternative pathway can be slowly activated by pathogen-associated molecular patterns (PAMPs) and damage-associated molecular patterns (DAMPs), but it usually sustains low-level complement system activation [48]. In contrast, the classical pathway is more likely to be activated in MASH, which can be triggered by antibody–antigen complexes of IgG or IgM [49]. However, evidence revealed that IgM played a protective role in chronic inflammation of the liver and insulin resistance [10,11]. Similarly, in the current study, the IgM of the MASH group was lower than that of the non-MASH group. On the other hand, IgG-immune complex has been reported to initiate the classical pathway to promote the production of C3 (C3a) [37,50] and elevated IgG was correlated with risk of hepatic decompensation in MASH patients [9]. Therefore, we hypothesized that the activation of C3 was dependent on IgG. By detecting serum IgG in the mouse model, we did find it increased in the HF diet-fed group.

Next, by using different assays, we demonstrated that, consistent with the above result of MASH patients, C3 was elevated in the OA+PA-treated cell model and HF diet-fed animal model. The same was true for the active fragment C3a. C3 activation marker C3a is associated with hepatic steatosis and hepatocyte injury in individuals with severe obesity [51]. Moreover, there were deposits of activated forms of C3 in the liver of 74% MASLD patients [44]. In particular, it has been found that severity of MASLD is associated with the C3 around fatty degenerated hepatocytes [52], but the specific mechanism is not clear. Therefore, we further explored how C3 affected the development of MASH.

As a central player in complement activation and immune defense, C3 can induce macrophages, hepatic stellate cells (HSCs), and neutrophils to generate a variety of immune responses. Meanwhile, the receptor of C3a (C3aR) is highly expressed on neutrophils [28]. C3a and neutrophil infiltration is associated with MASLD progression. C3a/C3aR may coactivate neutrophils with C5aR2, but the specific mechanism needs to be explored further [53]. Adding anti-C3a antibody to neutralize C3a inhibits the formation of NETs [54]. NETs are fibrous networks protruding from the membrane of activated neutrophils to promote phagocytosis of specific substances and then stimulate inflammation, which is consistent with the role of the complement system. Subsequently, we detected the formation of NETs in MASH: marker proteins of NETs, citH3, and MPO, had elevated expression in MASH model and patients. Levels of NETs-related genes were also high in the HF group. Moreover, the HF group and MASH patients had a higher rate of positive co-staining for NETs than the control group and healthy subjects. Similarly, NETs were found in 94.1% (16/17) of MASH biopsy specimens, while they were absent in the control specimens [55].

Furthermore, murine neutrophils were isolated and subjected to direct C3 intervention. Our findings demonstrated that C3 directly upregulated the expression of citH3 and NETs-associated genes, and triggered enhanced NET formation, as confirmed by extracellular DNA-MPO-CitH3 colocalization assays. In a liver biopsy study, the extensive deposition of C3 in liver of MASLD patients was related to excessive fat accumulation, hepatocyte apoptosis, and liver neutrophil isolation [44]. In vivo experiments have shown that mice with C3 knockout exhibited reduced neutrophil infiltration and NETs formation [17]. These results similarly suggested that C3 promoted NETs formation and C3 deficiency ameliorated cellular injury by reducing neutrophil infiltration. All in all, we propose a possible mechanism for the progression of MASH that elevates IgG-activated C3 to stimulate the production of NETs, thereby exacerbating MASH (Figure 5E). These findings would help in the prediction of MASLD and provide novel targets to prevent the disease.

Notably, the C3a receptor is also expressed on other immune cells, including macrophages, and HSCs. C3a-C3aR signaling mediated proinflammatory activation of macrophages [56] and enhanced IL1b activity in macrophages [57]. The C3-C3aR axis promoted macrophage recruitment, and in vitro migration assays demonstrated dose-dependent enhancement of macrophage migration [58,59]. However, macrophage-specific C3aR knockout mice exhibited no significant alterations in body weight, glucose metabolism, hepatic steatosis, or fibrosis compared to the diet-induced MASLD control group [60], suggesting a nonessential role of C3aR in macrophages in MASLD pathogenesis. For HSCs, their activation was upregulated in high C3 group of Schistosoma japonicum patients. Exogenous C3 administration induced hepatic structural damage and collagen deposition in murine models [61]. Additionally, single-cell RNA sequencing analyses of non-cirrhotic and cirrhotic livers identified C3 as a critical regulator driving HSC differentiation into myofibroblasts [62]. These findings indicated that C3-mediated effects may not be limited to NETs, potentially involving coordinated interactions with multiple immune cells. Further cell co-culture experiments and single-cell RNA sequencing will resolve the crosstalk between NETs and other immune effectors.

Moreover, while our current study focuses on the MASH pathogenesis, it is also vital to track IgG/C3/NETs kinetics in early steatosis. Emerging evidence suggests that IgG and C3 may already play roles in early MASLD stages. A retrospective cross-sectional study reported elevated IgG levels in 27.3% of MAFL patients and 47.7% of MASH patients, implying IgG’s involvement even in steatosis [63]. C3 levels rise significantly with MASLD severity (controls: 104.7 mg/dL; MASL: 142.3 mg/dL; MASH: 166.2 mg/dL) [64], and a meta-analysis confirms higher C3 in moderate/severe MASLD versus mild cases [65]. Prior work demonstrates that NETs are strongly associated with advanced MASLD, detected in 94.1% of MASH biopsies but absent in simple steatosis [55]. Mechanistically, NET inhibition, via DNase1 or PADI4 knockout, does not prevent FFA accumulation, whereas FFAs directly induce NET formation in vitro [66]. This supports the notion that NETs are downstream consequences of lipid overload, likely amplifying inflammation in later stages (e.g., MASH). These findings suggest that IgG and C3 may accumulate during early steatosis but may not yet reach thresholds required to drive NET formation or overt inflammation. Further studies are required to characterize IgG, C3, and NETs in a MASLD-only cohort and explore whether IgG–C3 interactions during steatosis prime the liver for subsequent NETs-driven injury in MASH.

There are some limitations to this study. Firstly, the clinical data were collected from a single-center cohort, and included predominantly low-fibrosis patients. Cirrhosis-associated immune dysfunction may alter immunoglobulin/complement profiles [67]. The findings may not fully extrapolate to advanced fibrosis/cirrhosis. Longitudinal cohorts from other sources and including advanced fibrosis/cirrhosis are needed. Secondly, there was no experimental validation regarding how IgG directly activates C3 in MASH, although prior studies have demonstrated that IgG activates C3 through the classical pathway. Further investigations (IgG-C3 co-immunoprecipitation or IgG blockade) are required to elucidate this interaction in MASH pathogenesis. In addition, IgA has the potential to be a biomarker for MASH, but its role with complement has not been clarified and needs to be explained by future studies. Finally, the current study mainly identified a correlation between C3 activation and MASH, whereas further functional studies in in vivo animal models (e.g., C3 knockout/overexpression models) are required to strengthen the important conclusions regarding C3 as an important driver and the causal relationship between C3 and MASH progression.

## 5. Conclusions

In summary, the ratio of ANA positivity was higher in the MASH group than that in non-MASH group. IgA and C3 correlated with the severity of MASLD. If IgA > 2.990 g/L or C3 > 1.115 g/L, the patient should be alerted to the presence of MASH. In addition, elevated IgG in MASH may activate C3 to stimulate the production of NETs, thus exacerbating the disease.

## Figures and Tables

**Table 1:** Comparison of characteristics between MASH group and non-MASH group.

Parameter	Non-MASH Group (NAS < 5, *N* = 65)	MASH Group (NAS ≥ 5, *N* = 21)	X^2^/T Value	*p* Value
Autoantibody (%)			2.747	0.097
positive	33 (50.8)	15 (71.4)		
negative	32 (49.2)	6 (28.6)		
ANA (%)			4.018	0.045
positive	27 (41.5)	14 (66.7)		
negative	38 (58.5)	7 (33.3)		
Different titers of ANA (%)			7.808	0.076
<1:100	38 (58.5)	7 (33.3)		
1:100	15 (23.1)	4 (19.0)		
1:320	8 (12.3)	7 (33.3)		
1:1000	2 (3.1)	1 (4.8)		
1:3200	2 (3.1)	2 (9.5)		
IgE (IU/mL)	25.8 [13.7, 58.8]	68.9 [16.3, 171.6]	−1.817	0.069
IgG (g/L)	12.4 ± 2.9	13.3 ± 4.4	−0.936	0.353
IgA (g/L)	2.4 ± 0.8	3.0 ± 1.2	−2.322	0.023
IgM (g/L)	1.1 [0.9, 1.5]	1.0 [0.8, 1.4]	0.179	0.858
Complement C3 (g/L)	0.9 ± 0.1	1.1 ± 0.2	−3.019	0.007
Complement C4 (g/L)	0.2 [0.2, 0.2]	0.2 [0.2, 0.3]	−2.060	0.039
ALT (U/L)	50.0 [30.0, 87.0]	94.0 [44.5, 152.5]	−2.128	0.033
AST (U/L)	36.0 [25.0, 56.0]	55.0 [38.5, 77.0]	−2.635	0.008

**Table 2:** Comparison of pathological features between autoantibody negative group and autoantibody positive group.

Parameter		Autoantibody Positive Group (*N* = 48)	Autoantibody Negative Group (*N* = 38)	X^2^/T Value	*p* Value
Steatosis				2.778	0.423
	<5%	3 (6.3)	6 (15.8)		
	5~33%	21 (43.8)	18 (47.4)		
	34~66%	14 (29.2)	9 (23.7)		
	>66%	10 (20.8)	5 (13.2)		
Ballooning				4.455	0.104
	None	16 (33.3)	21 (55.3)		
	Few balloon cells	20 (41.7)	12 (31.6)		
	Many cells/prominent ballooning	12 (25.0)	5 (13.2)		
Lobular inflammation (foci per 200× field)				2.747	0.224
	None	7 (14.6)	11 (28.9)		
	<2	35 (72.9)	24 (63.2)		
	2~4	6 (12.5)	3 (7.9)		
	>4	0	0		
NAS scores		3.54 ± 1.79	2.71 ± 1.69	2.194	0.031
Diagnosed as MASH (%)		15 (31.3)	6 (15.8)	2.747	0.097
Fibrosis				3.665	0.472
	F0	22 (45.8)	16 (42.1)		
	F1	12 (25.0)	16 (42.1)		
	F2	8 (16.7)	3 (7.9)		
	F3	2 (4.2)	1 (2.6)		
	F4	4 (8.3)	2 (5.3)		

**Table 3:** Multivariate analysis for NAS.

Variable	NAS ≥ 5 vs. <5	
	OR (95% CI)	*p* Value
IgA	2.403 (1.096–5.267)	0.029
IgG	0.848 (0.673–1.069)	0.164
Complement C3	73.372 (1.108–4857.767)	0.045
ANA	1.627 (0.873–3.034)	0.126

CI: Confidence interval.

## Data Availability

The original contributions presented in this study are included in the article. Further inquiries can be directed to the corresponding authors.

## Supplementary Materials

The following supporting information can be downloaded at https://www.mdpi.com/article/10.3390/cells14100740/s1, Figure S1: ROC curves for complement C3, IgA, and their combination to assess whether NAS scores are more than 4 in MASLD patients; Table S1: The primer sequences; Table S2: Demographic and clinical characteristics of MASLD patients; Table S3: Pathological characteristics of MASLD patients; Table S4: Comparison of clinical features between autoantibody negative group and autoantibody positive group; Table S5: Univariate analysis with steatosis and ballooning; Table S6: Univariate analysis with lobular inflammation and NAS; Table S7. Association of the genes in Figure 3E with NETs and their expression in RNA sequencing. References [68,69,70,71,72,73,74,75,76,77,78,79,80,81,82,83,84,85,86,87,88,89,90,91] are cited in the Supplementary Materials.

## Abbreviations

The following abbreviations are used in this manuscript:

AIH	Autoimmune hepatitis
AMA	Anti-mitochondrial antibody
ANA	Antinuclear antibodies
AUC	Area under curve
BMI	Body mass index
C3aR	Receptor of C3a
CAP	Controlled attenuation parameter
CI	Confidence interval
citH3	Citrullinated histone H3
CSF3	Colony-stimulating factor 3
CXCL1	C-X-C motif chemokine ligand 1
DAMPs	Damage-associated molecular patterns
FFA	Free fatty acid
GAN	Gubra amylin NASH
HE	Hematoxylin-eosin
HF	High-fat
HSCs	Hepatic stellate cells
IgG	Immunoglobulin G
IgM	Immunoglobulin M
IL1b	Interleukin-1b
LKM	Liver-kidney microsome antibody
LSM	Liver stiffness measurements
MASLD	Metabolic dysfunction-associated steatotic liver disease
MASH	Metabolic dysfunction-associated steatohepatitis
MPO	Myeloperoxidase
NAS	Non-alcoholic fatty liver disease activity score
NETs	Neutrophil extracellular traps
OA	Oleic acid
PA	Palmitic acid
PADI4	Peptidyl arginine deiminase 4
PAMPs	Pathogen-associated molecular patterns
RBC	Red blood cell count
ROC	Receiver operating characteristic
SEM	Standard error of mean
SMA	Smooth muscle antibody
TNFa	Tumor necrosis factor a
