# Supplementary material for: Activated Immune and Complement C3 Are Potential Contributors in MASH via Stimulating Neutrophil Extracellular Traps
Source: Cells. 2025 May 19;14(10):740. doi: 10.3390/cells14100740 (PMC12110206; doi:10.3390/cells14100740)

Supplementary Table S1. The primer sequences

Gene	Primer F	Primer R
C3	CCAGCTCCCCATTAGCTCTG	GCACTTGCCTCTTTAGGAAGTC
TNFa	CAGGCGGTGCCTATGTCTC	CGATCACCCCGAAGTTCAGTAG
CSF3	GCACTATGGTCAGGACGAGAG	GGGGAAATACCCGATAGAGCC
IL1b	GAAATGCCACCTTTTGACAGTG	TGGATGCTCTCATCAGGACAG
PADI4	TGGTCCTCCAGTCAAGAAGAG	GCTTTCACCTGTAGGGTCACC
MPO	AGTTGTGCTGAGCTGTATGGA	CGGCTGCTTGAAGTAAAACAGG

Supplementary Table S2. Demographic and clinical characteristics of MASLD patients.

Parameter		Mean or median values (N = 182)
Demographic characteristics	Age (years)	42.4±15.6
	Male (%)	104(57.1)
	BMI (kg/m²)	25.8[23.2,28.7]
Comorbidities	Hypertension (%)	74(40.7)
	Diabetes (%)	36(19.8)
Autoantibody test	At least one positive test (%)	67(36.8)
	ANA positive (%)	57(31.3)
Different titers of ANA	＜1:100	125(68.7)
	1:100	33(18.1)
	1:320	16(8.8)
	1:1000	3(1.6)
	1:3200	5(2.7)
Immunoglobulin	IgE(IU/ml)	35.6[16.9,86.7]
	IgG(g/L)	12.0±3.1
	IgA(g/L)	2.4[1.6,2.9]
	IgM(g/L)	1.1[0.8,1.5]
Complement	Complement C3(g/L)	1.0±0.2
	Complement C4(g/L)	0.2[0.2,0.2]
Biochemical tests	Total protein(g/L)	69.0±6.6
	Albumin(g/L)	43.8[40.7,47.4]
	Prealbumin (g/L)	0.2±0.1
	ALT(U/L)	56.0[30.5,108.0]

	AST(U/L)	39.0[25.5,57.0]
	ALP(U/L)	79.0[61.0,104.0]
	R-GT(U/L)	55.0[29.5,104.0]
	Direct bilrubin (umol/L)	5.0[3.6,6.8]
	Total bilrubin (umol/L)	13.8[10.4,18.7]
	Uric acid (umol/L)	375.5[297.5,455.2]
	Urea nitrogen (mmol/L)	4.4[3.7,5.4]
	Creatinine (umol/L)	66.7[55.8,77.6]
	Calcium (mmol/L)	2.3[2.2,2.4]
Blood lipids	TC(mmol/L)	4.7±1.0
	TG(mmol/L)	1.6[1.1,2.2]
	LDL(mmol/L)	2.9±0.8
	HDL(mmol/L)	1.1±0.3
Coagulation	PT(s)	12.7[12.3,13.3]
	APTT(s)	36.5[34.4,38.6]
	TT(s)	17.5[16.6,18.6]
Blood tests	Red Blood Cell Count (10^12/L)	4.6[4.2,5.0]
	Hemoglobin (g/L)	140.0[128.0,153.0]
	Hematocrit (%)	41.7[38.6,45.0]
Fibrotouch	Controlled Attenuation parameter (db/m)	277.0[245.0,308.5]
	Liver stiffness measurernents (kpa)	8.0[6.3,11.9]

Supplementary Table S3. Pathological characteristics of MASLD patients

	Parameter	Number (N = 86)
Steatosis	＜5%	9(10.5)
	5%～33%	39(45.3)
	34%～66%	23(26.7)
	＞66%	15(17.4)
Ballooning	None	37(43.0)
	Few balloon cells	32(37.2)
	Many cells/prominent ballooning	17(19.8)
Lobular inflammation (foci per 200×field)	None	18(20.9)
	<2	59(68.6)
	2～4	9(10.5)
	>4	0

NAS scores		3.2±1.8
Diagnosed as MASH (%)		21(24.4)
Fibrosis	F0	38(44.2)
	F1	28(32.6)
	F2	11(12.8)
	F3	3(3.5)
	F4	6(7.0)

Supplementary Table S4. Comparison of clinical features between autoantibody negative group and autoantibody positive group

Parameter		Autoantibody positive group （N = 67）	Autoantibody negative group （N = 115）	*p* value
Demographic characteristics	Age (years)	43.2±16.1	41.8±15.3	0.568
	Male (%)	23(34.3)	81(70.4)	0.000
	BMI (kg/m²)	24.7[22.4,28.2]	26.1[23.6,29.1]	0.052
Comorbidities	Hypertension (%)	24(35.8)	50(43.5)	0.310
	Diabetes (%)	11(16.4)	25(21.7)	0.385
Immunoglobulin	IgE(IU/mL)	36.44[16.28,83.20]	33.73[17.26,111.05]	0.735
	IgG(g/L)	13.1±3.7	11.0±2.0	0.000
	IgA(g/L)	2.485[1.733,3.008]	2.135[1.530,2.950]	0.148
	IgM(g/L)	1.170[0.933,1.733]	0.949[0.778,1.305]	0.007
Complement	C3(g/L)	0.983±0.224	0.990±0.238	0.859
	C4(g/L)	0.194[0.171,0.249]	0.200[0.172,0.252]	0.552
Biochemical tests	Albumin(g/L)	43.3[40.3,47.4]	44.0[41.1,47.4]	0.441
	Prealbumin (g/L)	0.246±0.068	0.266±0.584	0.053
	ALT(U/L)	58.0[29.0,121.0]	52.5[31.0,92.5]	0.559
	AST(U/L)	45.0[29.0,59.0]	36.0[25.0,57.0]	0.207
	ALP(U/L)	78.0[60.0,96.0]	85.0[63.7,107.2]	0.273
	r-GT(U/L)	54.0[28.0,106.0]	56.0[32.7,103.5]	0.316
	Direct bilrubin (umol/L)	4.9[3.3,6.6]	5.0[3.6,7.0]	0.663
	Total bilrubin (umol/L)	13.8[10.2,19.1]	13.9[10.7,18.7]	0.475
Blood lipids	TC(mmol/L)	4.6±1.0	4.8±1.0	0.184
	TG(mmol/L)	1.4[1.1,2.1]	1.6[1.1,2.4]	0.141
	LDL(mmol/L)	2.8±0.9	3.0±0.8	0.133
	HDL(mmol/L)	1.1±0.3	1.1±0.3	0.827
FibroTouch	Controlled Attenuation	273.0[244.5,295.5]	283.0[245.0,310.5]	0.393

parameter (db/m)			
Liver stiffness measurernents (kpa)	8.5[6.7,11.4]	7.6[5.9,12.1]	0.436

Supplementary Table S5. Univariate analysis with steatosis and ballooning

Parameter	Steatosis >33%VS≤33%		Ballooning: Many cells/prominent ballooning VS None or few balloon cells	
	OR (95% CI)	*P* value	OR (95% CI)	*p* value
IgE	1.001(0.998-1.003)	0.644	1.002(0.999-1.005)	0.137
IgG	0.997(0.862-1.153)	0.967	1.080(0.908-1.285)	0.383
IgA	2.094(1.100-3.987)	0.024	1.531(0.835-2.809)	0.168
IgM	0.580(0.223-1.509)	0.265	0.648(0.180-2.330)	0.507
Complement C3	76.728(3.058-1924.918)	0.008	453.865(8.795-23421.296)	0.002
Complement C4	370.313 (0.057-2410625.066)	0.187	15904.458 (0.299-847382923.1)	0.081
ANA	1.324(0.895-1.959)	0.160	1.724(1.102-2.697)	0.017
Autoantibody positve	0.552(0.233-1.309)	0.177	0.382(0.122-1.201)	0.100
ALT	1.006(1.000-1.013)	0.047	1.001(0.994-1.008)	0.734
AST	1.004(0.996-1.012)	0.320	0.999(0.989-1.008)	0.800

Supplementary Table S6. Univariate analysis with lobular inflammation and NAS

Parameter	Lobular inflammation (foci per 200×field) Yes VS No		NAS ≥ 5 VS < 5	
	OR (95% CI)	*P* value	OR (95% CI)	*p* value
IgE	0.999(0.997-1.002)	0.651	1.002(0.999-1.006)	0.175
IgG	1.084(0.868-1.353)	0.478	1.080(0.919-1.269)	0.351
IgA	2.195(0.855-5.631)	0.102	1.938(1.044-3.598)	0.036
IgM	0.460(0.163-1.296)	0.142	0.719(0.239-2.161)	0.557
Complement C3	0.855(0.025-28.721)	0.930	361.089(8.831-14763.807)	0.002
Complement C4	0.000(0.000-25.247)	0.155	1356.891(0.074-24994435.21)	0.150
ANA	1.245(0.740-2.094)	0.409	1.669(1.088-2.561)	0.019
Autoantibody positve	0.372(0.125-1.107)	0.076	0.456(0.163-1.276)	0.135
ALT	1.007(0.997-1.016)	0.170	1.005(0.999-1.011)	0.137
AST	1.018(0.997-1.040)	0.100	1.001(0.994-1.009)	0.699

Supplementary Table S7. Association of the genes in Figure 3E with NETs and their expression in RNA sequencing

Genes	Description (coding protein)	Association with NETs	Control group	HF group	*p* value
AKT1	AKT serine/threonine kinase 1	Elicits a switch from neutrophil apoptosis to NETs[68]	17.432±0.444	18.593±2.198	0.614
AKT2	AKT serine/threonine kinase 2	Promotes extracellular DNA release[69]	16.106±0.575	15.531±0.682	0.531
ATG7	Autophagy related 7	Promotes peptidyl arginine deiminase 4 expression, and inducing the formation of NETs[70]	7.966±0.376	9.454±0.769	0.108
CSF3	Granulocyte colony stimulating factor	Modulates neutrophil recruitment/activation[71]	0±0	0±0	
CTSG	Cathepsin G	A component of NETs[72]	0.047±0.027	0±0	0.106
CYBB	NADPH oxidase	Encodes NOX2 subunit of NADPH oxidase, essential for ROS production driving NETs[73]	4.29±0.644	6.349±2.14	0.375
DNASE1	Deoxyribonuclease I	Degrades extracellular DNA in NETs post-release to prevent pathological tissue damage[74]	0.336±0.051	0.399±0.077	0.512
ELANE	Neutrophil elastase	Neutrophil elastase critical for histone cleavage and subsequent chromatin decompaction[75]	0.11±0.062	0.384±0.056	0.007
ENTPD4	Ectonucleoside Triphosphate Diphosphohydrolase 4	Modulates extracellular ATP/ADP balance to regulate neutrophil activation thresholds[76]	4.232±0.448	5.572±0.455	0.058
F3	Coagulation Factor III,	Combines with DNA fibers or nuclear	1.463±0.185	1.932±0.579	0.455

	tissue factor	chromatin to form NETs[77]			
HMGB1	High mobility group box 1	Promotes neutrophil recruitment and activation[78]	16.71±0.587	17.646±1.289	0.521
IL1B	Interleukin 1 beta	Induces NETs via NLRP3 inflammasome activation and pyroptosis cross-talk[79]	0±0	0±0	
IL6	Interleukin 6	Promotes neutrophil recruitment and activation[80]	1.393±0.104	2.622±0.427	0.016
IL17A	Interleukin 17	Pro-inflammatory cytokine enhancing NETs formation[81]	0.036±0.036	0.019±0.012	0.658
IRAK4	Interleukin 1 receptor associated kinase 4	Involved in Toll-like receptor and IL-1R signaling pathways[82]	2.386±0.114	3.711±0.281	0.001
ITGAM	Complement component 3 receptor 3 subunit	Cooperates with ITGB2 to mediate neutrophil extracellular matrix interactions preceding NETs release[83]	0.247±0.038	0.61±0.276	0.218
ITGB2	Complement component 3 receptor 3 and 4 subunit	β2 integrin subunit required for neutrophil adhesion and cytoskeletal remodeling during NETs extrusion[84]	3.566±0.519	6.641±1.888	0.142
KCNN3	Potassium channel, calcium activated	Mediates NADPH oxidase-independent NETs induced by calcium influx[85]	0±0	0.004±0.003	0.159
MAPK1	Mitogen-activated protein kinase 1	Transduces pro-inflammatory signals activating transcriptional regulators of NETs[86]	15.792±0.56	16.827±0.929	0.359
MAPK3	Mitogen-activated protein kinase 3	Transduces pro-inflammatory signals activating transcriptional	10.855±0.394	14.395±3.042	0.271

		regulators of NETs			
MMP9	Matrix metallopeptidase 9	Metalloproteinase facilitating extracellular matrix remodeling to enable NETs expansion[79]	0.274±0.047	0.394±0.095	0.277
MPO	Myeloperoxidase	A component of neutrophil granules containing antimicrobial substances[79]	0.081±0.053	0.013±0.013	0.237
MTOR	Mechanistic target of rapamycin kinase	Modulated by HMGB1 to stimulate NETs formation[87]	4.374±0.205	4.326±0.259	0.886
PADI4	Peptidyl arginine deiminase 4	Catalyzes histone citrullination, neutralizing histone-DNA interactions to enable chromatin decondensation[77]	0.011±0.011	0.07±0.029	0.079
PTAFR	Platelet activation factor receptor	Mediates neutrophil-platelet interactions that potentiate NETs[79]	0.138±0.016	0.74±0.268	0.045
PIK3CA	Phosphatidylinositol-4,5-bisphosphate 3-kinase	Activates AKT/mTOR signaling axis to regulate NETs progression	3.02±0.154	2.675±0.201	0.199
RIPK1	Receptor interacting serine/threonine kinase 1	Necroptosis pathway component that synergizes with NETs[88]	6.157±0.268	5.907±0.582	0.704
RIPK3	Receptor interacting serine/threonine kinase 3	Necroptosis pathway component that synergizes with NETs	0.512±0.051	0.864±0.093	0.006
SELP	P-selectin	Binds to PSGL-1 on neutrophils to induce NETs formation[89]	1.081±0.099	1.382±0.086	0.041
SELPLG	P-selectin receptor	A receptor on neutrophils to induce NETs formation[89]	1.972±0.244	3.823±1.293	0.185
TLR2	Toll like receptor 2	Pathogen-sensing receptor that triggers	0.913±0.087	1.936±0.511	0.072

		NETs formation through signaling cascades[90]			
TLR4	Toll like receptor 4	Pathogen-sensing receptor that triggers NETs formation through signaling cascades[91]	0.438±0.07	0.616±0.146	0.291
TLR7	Toll like receptor 7	Pathogen-sensing receptor that triggers NETs formation through signaling cascades	0.978±0.225	1.149±0.441	0.737
TLR8	Toll like receptor 8	Pathogen-sensing receptor that triggers NETs formation through signaling cascades	0.584±0.117	0.725±0.296	0.664
TNF	Tumor necrosis factor-alpha	Primes neutrophils for enhanced ROS production and accelerates NETs formation[79]	0.047±0.014	0.303±0.115	0.047

Supplementary Figure S1 ROC curves for complement C3, IgA, and their combination to assess whether NAS scores are more than 4 in MASLD patients.

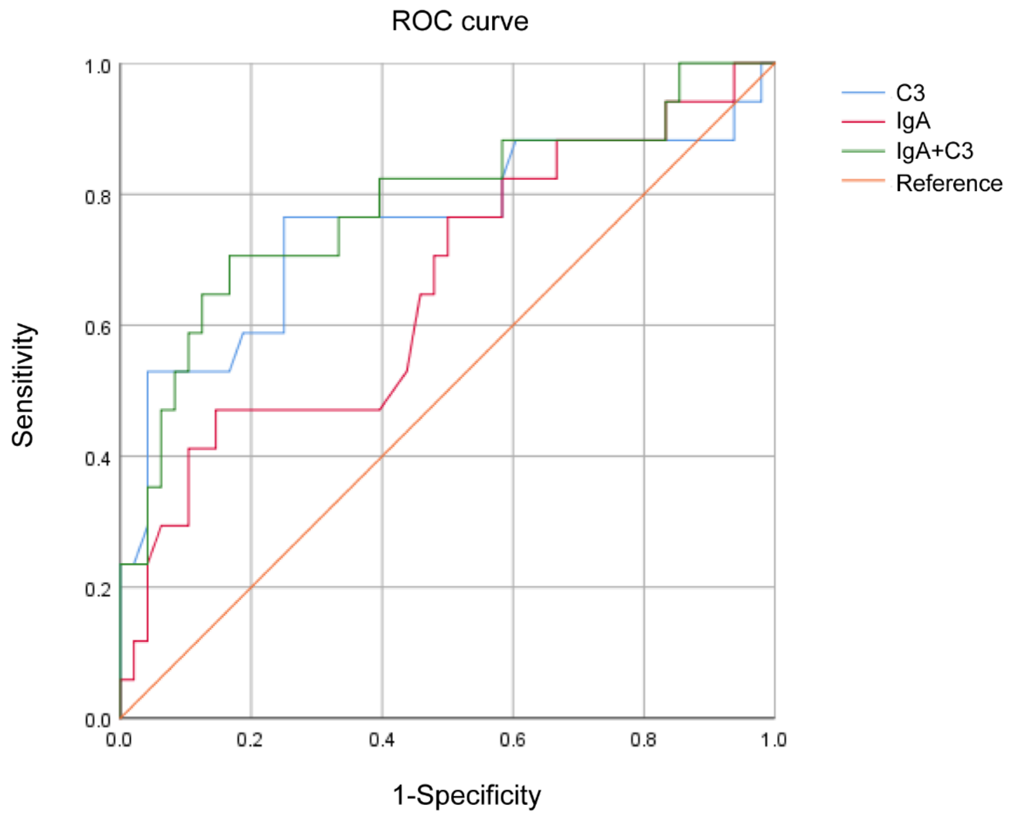

Supplement: Supplementary file 1 [file cells-14-00740-s001.zip › cells-3592536-supplementary.pdf]
